# Sex-specific lung functional changes in adult mice exposed only to second-hand smoke *in utero*

**DOI:** 10.1186/s12931-017-0591-0

**Published:** 2017-06-27

**Authors:** Alexandra Noël, Rui Xiao, Zakia Perveen, Hasan Zaman, Viviana Le Donne, Arthur Penn

**Affiliations:** 1 Department of Comparative Biomedical Sciences, School of Veterinary Medicine, Louisiana State University, Skip Bertman Drive, Baton Rouge, 70803 LA USA; 2 Department of Anesthesiology, Columbia University Medical Center, 622 West 168th Street, New York, 10032 NY USA; 3 Translational Medicine and Comparative Pathobiology, R&D Platform Technology and Science, GlaxoSmithKline, Park Road, Ware, SG12 ODP UK

**Keywords:** *In utero* exposures, Second-hand smoke, Lung function, Lung structure, Sex specificity

## Abstract

**Background:**

An increasing number of epidemiological and experimental studies have associated exposure to second-hand smoke (SHS) during pregnancy with adverse outcomes in newborns. As we have previously shown in mice, *in utero* exposure to SHS at critical stages of fetal development, results in altered lung responses and increased disease susceptibility upon re-exposure to irritants (SHS or ovalbumin) in adulthood. In this study, we asked whether the *in utero* SHS exposure alone is sufficient to alter lung structure and function in adult mice.

**Methods:**

Pregnant BALB/c mice were exposed from days 6 to 19 of pregnancy to 10 mg/m^3^ of SHS or HEPA-filtered air. Male and female offspring (*n* = 13–15/group) were sacrificed at 15 weeks of age. We measured lung function with non-invasive and invasive methods, performed lung morphometric analysis on trichrome-stained lung tissue samples, and assessed lung gene expression via RNA sequencing and protein assays.

**Results:**

*In utero* SHS exposure significantly increased mean linear intercept and decreased the surface area per unit volume of the lungs in both males and females, indicating perturbation in alveolar developmental processes. Tidal volume, minute volume and inspiratory capacity were significantly decreased compared with the controls only in male mice exposed *in utero* to SHS, suggesting that males are more sensitive than females to an SHS insult during lung development. This also suggests that in our model, lung structure changes may be necessary but are not sufficient to impair lung function. SERPINA1A, the mouse ortholog of human α1-antitrypsin, deficiency of which is a known genetic risk factor for emphysema, was down-regulated at the protein level in the *in utero* SHS-exposed mice. Additionally, DNMT3A protein expression was dysregulated, indicating that DNA methylation occurred in the lungs.

**Conclusions:**

Our results indicate that *in utero* SHS exposure alone alters both lung function and structure well into adulthood (15 weeks) in male mice. Furthermore, lung function alterations in this model are sex-specific, with males being more susceptible to *in utero* SHS effects. Overall, our data suggest that *in utero* SHS exposure alone can predispose to adult lung diseases.

## Background

Since the first Surgeon General’s Report on Smoking and Health in 1964 [1], tobacco use has been recognized as a leading public health concern. Currently, over 5 million deaths worldwide occur annually due to direct tobacco use, with second-hand smoke (SHS) exposures being responsible for approximately 600,000 of those deaths [1, 2]. In 1994, the US EPA classified SHS as a class A carcinogen. SHS aerosols are a complex mixture of highly toxic particles and gases that contain more than 4000 different chemicals, including polynuclear aromatic hydrocarbons (PAHs), 250 cytotoxic compounds and at least 50 substances that are classified as known or possibly carcinogenic to humans, such as formaldehyde, cadmium, nickel and benzo(a)pyrene [2–4]. Annually, in the United States, over 126 million people, including pregnant women and women of childbearing age, are exposed to SHS [5]. This is in addition to the 10% of women who smoke during their pregnancy [6]. Thus, unborn children are a vulnerable subpopulation involuntarily exposed to cigarette smoke and SHS. It is now well documented that active smoking during pregnancy causes altered lung function, including decreased compliance and expiratory flow rates, as well as increased resistance, and increased lower respiratory diseases in the offspring [7–12]. However, few studies have investigated the contribution of *in utero* SHS exposures to these same outcomes. Currently, there is a growing body of epidemiological evidence showing that *in utero* exposures to SHS can affect fetal development and result in adverse effects ranging from low birth weight to increased disease susceptibility in adulthood [13–15].

As early as 1967, the health consequences of SHS to children, particularly on the respiratory tract, were recognized and documented [16]. More recent studies have shown that *in utero* exposure to environmental pollutants, including SHS, results in alterations of physical and physiological features of the fetus, as well as increased disease susceptibility in adulthood [17–20]. Studies from our lab and others have shown that *in utero* SHS exposure exacerbates adult responses to environmental stressors, including house dust mites, *Aspergillus fumigatus* and SHS [18, 21, 22]. We have previously established that *in utero* exposure to SHS aggravates airway hyperresponsiveness, and increases expression of chemokines, cytokines, and acute phase response genes, which results in establishment of a pro-asthmatic milieu in ovalbumin-challenged adult mice [20, 23]. We also showed that upon re-exposure to SHS as adult, mice exposed *in utero* to SHS exhibited altered lung structure [21]. Overall, we have demonstrated that *in utero* exposure to SHS 1) elicits persistent well-documented pathophysiological and molecular changes in various adult lung disease models, upon re-exposure to an irritant as an adult, and 2) modulates responses related to lung function and structure, as well as transcriptomic alterations. Meanwhile, epidemiological studies associated *in utero* exposure to cigarette smoke with sustained lung deficiencies, which could lead to impaired lung function following additional postnatal exposures [8, 24–26]. Moreover, epidemiological studies have demonstrated that *in utero* SHS exposures are a risk factor for pulmonary diseases, including asthma, as well as for altered lung function [27, 28]. Despite those clear associations, little is known about whether *in utero* SHS exposure alone permanently alters lung function and/or structure in the offspring. This is due mostly to the fact the human lung is exposed to a multitude of irritants throughout life, e.g. allergens and bacteria, making the contribution of *in utero* SHS exposure alone on lung health challenging to assess [29–31]. Thus, there is still insufficient evidence to define the long-term contribution of *in utero* SHS exposure alone, as a sole or baseline factor, to modification of lung function and structure, thereby predisposing to the development of lung diseases, such as asthma, emphysema, chronic bronchitis, fibrosis or cancer [29–31]. To address this knowledge gap, in the present study we asked whether *in utero* SHS exposure alone, without any postnatal re-exposure to an irritant, is sufficient to alter lung structure and function in adult 15-week old mice.

## Methods

### Animal protocols and SHS exposures

**Fig. 1:**
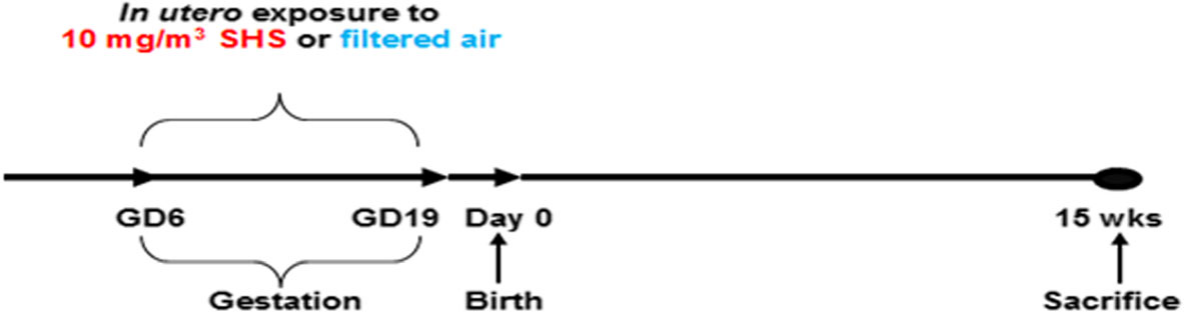
Experimental study design

We conducted SHS exposures on BALB/c mice (Harlan, Indianapolis, IN), as described previously [20, 21, 23]. The experimental study design is presented in Fig. 1. SHS is composed of approximately 90% sidestream smoke and 10% mainstream smoke [32]. In this study, mice were exposed to 100% sidestream smoke, which is used as a surrogate for SHS. Briefly, beginning on the 6^th^ day after mating, half of the mated females (*n* = 27), randomly selected, were exposed to SHS generated from 3R4F filtered research cigarettes (University of Kentucky, Lexington, KY). The SHS was mixed with HEPA-filtered air to reach a total particulate matter concentration of 10 mg/m^3^. Exposures lasted 4 h per day from days 6 to 19 of gestation. The SHS was generated by a 30-port cigarette smoke machine (AMESA Technologies, Geneva, Switzerland). Atmosphere samples were collected for gravimetric analysis at a flow rate of 3 L/min throughout the experiment on a cassette holding a 25 mm hydrophilic glass fiber filter with a 0.7 μm pore size (Millipore, AP4002500). The mass concentrations were followed and adjusted in real time using a DustTrak Aerosol Monitor 8520 (TSI Inc., Shoreview, MN, USA) previously calibrated with the SHS aerosol. The remaining mated females (*n* = 28) were exposed to 100% HEPA-filtered air. All exposures (HEPA-filtered air and SHS) occurred in 1.3 m^3^ dynamic chambers with approximatively 15 air changes/hour. Table 1 shows the characterization of the SHS and control HEPA-filtered air exposures. SHS-exposed female mice (*n* = 27) gave birth to 18 litters (5.39 ± 2.4 (SD) pups/litter), with a male to female ratio of 0.60. Air-exposed female mice (*n* = 28) gave birth to 18 litters (5.39 ± 2.2 (SD) pups/litter), with a male to female ratio of 0.56. After weaning, a subgroup of these offspring was randomly distributed (4 or 5 per cage) into their respective groups: air *in utero* females (AF), air *in utero* males (AM), SHS *in utero* females (SF), SHS *in utero* males (SM). Each group contained 13 to 15 offspring (Table 2); the remaining offspring were used in other studies. From birth to sacrifice (at 15 weeks of age) mice were maintained on HEPA-filtered air ventilated racks. Mice were housed and handled in accord with the NIH Guide for the Care and Use of Laboratory Animals. All procedures and protocols were approved by the Louisiana State University Institutional Animal Care and Use Committee.

**Table 1:** Second-hand smoke exposure characterization

Parameters	Controls (AIR)	*In utero* 3R4F SHS
Mass concentration (mg/m^3^)^a^	–	11.70 ± 2.93
Number concentration (#/cm^3^)^b, c^	–	972 231
Mean geometric diameter (nm)^b, c^	–	29.2 ± 2.7
Geometric standard deviation^b, c^	–	2.9 ± 0.1
Temperature (°C)	23.7 ± 1.5	24.4 ± 1.7
Relative humidity (%)	41.7 ± 11.4	40.0 ± 13.9

*Abbreviations*: *SD* standard deviation

^a^Average mass concentration determined gravimetrically

^b^Measurements made with a scanning mobility particle sizer (SMPS)

^c^Values reported are averages from samples taken over 9 days

**Table 2:** Weight at sacrifice of all experimental groups

Experimental *in utero* exposed groups	*n*	Weight at 15 weeks of age (g) ± SD
Air males	13	28.34 ± 1.2
SHS males	15	28.07 ± 2.4
Air females	14	23.56 ± 1.8
SHS females	14	23.45 ± 1.3

### Bronchoalveolar lavage cell and fluid collection

As previously described [20], following euthanasia, we lavaged the lungs of each mouse that was not flexiVented (*n* = 7–8 per group) 2 times with 0.5 mL warm phosphate-buffered saline (PBS) and resuspended the centrifuged bronchoalveolar lavage (BAL) cell pellet in PBS for BAL differential counts (300 cells). Smears were stained with a modified Wright’s stain. The BAL supernatant was stored at −80 °C for subsequent analyses.

### Histopathology analysis of lungs

The lungs of the mice that were not flexiVented (*n* = 7–8 per group) were inflated and pressure-fixed (25 cm) with buffered formalin (10%) administered by intratracheal instillation. We followed previously published procedures [20, 21, 23] for sectioning and staining 5-mm thick lung sections. We performed trichrome staining on 5-mm thick paraffin-embedded tissue sections.

### Lung morphometric analysis

Lung morphometric analysis was based on previously published procedures [33]. In brief, a grid line system with 16 (4X4) horizontal lines was overlaid on each of 40X lung micrographs. The number of times a line intercepts an alveolar wall was used to calculate mean linear intercept (L_m_) and surface area per unit volume (SApUV) [32]. In total, 15 sections were analyzed per sample, with *n* = 5 samples per group. Images were obtained with a Nikon Eclipse E400 microscope (Nikon, Tokyo, Japan).

### Pulmonary function testing

#### Whole body plethysmography

At 15 weeks of age, we placed the mice into individual chambers of a whole body plethysmograph (Buxco, Troy, NY) and measured baseline tidal and minute volumes, as previously described [20, 21, 23]. The lung responses were recorded and readings over 5 min were averaged for each mouse (*n* = 8 per group).

#### flexiVent

At 15 weeks of age, pulmonary function were also measured via an invasive method (*n* = 6–7 per group). Mice were anesthetized by a ketamine/xylazine cocktail, tracheostomized, and placed in the forced oscillation measurements flexiVent system (SCIREQ, Montreal, Canada). Measurements were accepted only if the coefficient of determination was > 0.95. For each mouse, five measurements per parameter were averaged. At baseline, lung resistance (R), compliance (C) and inspiratory capacity (IC) were calculated using the single compartment model. Mice were euthanized by an intraperitoneal injection of Beuthanasia-D (Schering-Plough, NJ) following the procedure.

### Lung harvest and mRNA extraction

The lungs of the mice that were not flexiVented (*n* = 7–8 per group) were harvested and used for mRNA extraction. We followed previously described procedures [20, 21, 23], including RNA sample quantity and purity assessment with a NanoDrop ND-1000 Spectrophotometer (NanoDrop, Wilmington, DE), and further assayed 1:5 dilutions of RNA samples with an Agilent 2100 BioAnalyzer and Agilent RNA 6000 Nano Series II Kits (Agilent Technologies, Palo Alto, CA). All samples fell into the following ranges: 260/280 ratio: 1.97–2.09; 260/230 ratio: 2.19–2.52; RNA concentration: 0.43–1.12 μg/μl; RNA integrity number: 9.3–9.6.

### Gene expression analysis

We assessed global gene expression in lungs of individual 15-week-old male mice (*n* = 4 per group). Samples were processed by Expression Analysis (Morrisville, NC) and sequenced, by HiSeq - 2×50bp-PE sequencing, on an Illumina sequencing platform. Post-sequencing bioinformatics analysis was performed. Across all samples, the median number of actual reads was 29.9 million with 29 million on-target reads, after removal of sequencing artifacts. Our mouse lung transcriptome had a median of 16,466 genes and 31,072 isoforms that were detected. We converted RNA samples into cDNA libraries via the Illumina TruSeq Stranded mRNA sample preparation kit (Illumina # RS-122-2103). We considered gene probes with at least 2-fold up-/down-regulation (*p* < 0.05) and false discovery rate (FDR) < 0.05 to be differentially expressed. We analyzed gene expression clusters with DAVID [34].

### Extraction of proteins

We extracted proteins from frozen lung tissue (stored at −80 °C) as described [35]. Briefly, tissues placed inside aluminum foil were snap-frozen in liquid nitrogen, mechanically broken by light hammering, and quickly placed into 2 mL round bottom micro-centrifuge tubes containing 300 μL of RIPA lysis buffer (Santa Cruz Biotechnology, Dallas, TX, USA), and three 2.5 mm zirconia/silica beads (Biospec Products Inc.). Tissues were then lysed completely with the aid of Tissue Lyser II (Qiagen, Germantown, MD, USA) at 25 MHz for 2 min. The lysed tissue was centrifuged at 13,000 g for 5 min at 4 °C. Proteins in the supernatants were aliquoted and stored at −80 °C. We used BCA protein assay kits (Thermo Scientific, Waltham, MA, USA) to determine protein concentrations in the lung supernatants.

### Western blotting

We incubated 15 μg of protein extracts from each of 12 lung tissues (6 controls exposed to HEPA-filtered air and 6 mice exposed to SHS) in Laemmli buffer (Bio-Rad Laboratories Inc., Hercules, CA, USA) with 2-mercaptoethanol, at 95 °C for 5 min in a PCR machine, and resolved protein bands by SDS-PAGE on Any kD™ Mini-PROTEAN® TGX™ Gel (Bio-Rad) at 150 V. We used a 250-10 kDa pre-stained protein ladder (Bio-Rad, Precision Plus Protein™ Dual Color Standards, Cat# 161-0374) as a standard to monitor migration of protein on the gel. Proteins resolved on the gels were transferred onto PVDF membranes (Immobilon-P, pore size of 0.45 μm, Millipore Inc., USA) by the Trans-Blot Turbo™ Transfer System (Bio-Rad). Antibodies to ERK5 or MAPK7 (Cat. No. 3552) and β-actin (ACTB, Cat. No. 4967 L), as the control, were purchased from Cell Signaling Technology Inc., (Danvers, MA, USA). Antibodies to cytochrome DNMT3A (Cat No. PA-1-882), and SERPINA1A (Cat. No. PA5-1661) were obtained from Thermo-Fisher Scientific Inc., (Waltham, MA, USA). Antibodies to PHF1 (Cat. No. Ab184951) was obtained from Abcam (Cambridge, MA, USA). All antibodies were diluted in blocking buffer made with 1× Tris-Buffered-Saline (Bio-Rad) supplemented with 1% bovine serum albumin (Immucor Inc., Norcross, GA), and 0.1% Tween 20 (Bio-Rad). Immunoblots were detected with the aid of the ECL-Prime Western Blotting Detection reagent (GE Healthcare, UK). Western blot images were captured by with ChemiDoc™ Touch imaging system (Bio-Rad). The captured images were analyzed with Image Lab 5.2 (Bio-Rad). Experiments were run in duplicate or triplicate.

### Statistical procedures

Mouse weights, BALF cytology, lung function and lung morphometry were analyzed by the Student *t*-test for pairwise comparisons or by ANOVA followed by the Tukey’s test for multiple comparisons. Results were considered statistically significance at *p* < 0.05. We carried-out statistical analyses with the Statistical Package for the Social Sciences (SPSS, version 17.0, SPSS Inc.).

## Results

### *In utero* SHS exposure alone alters lung structure in male and female mice

**Fig. 2:**
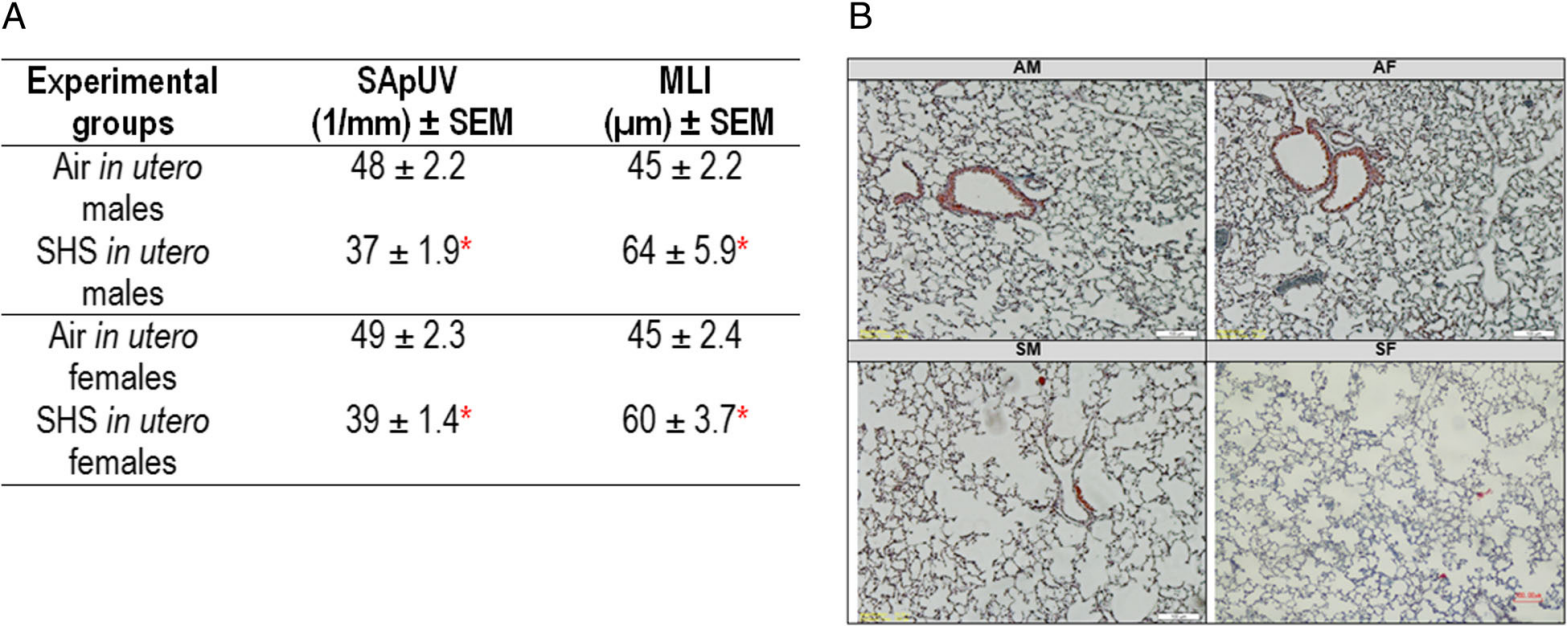
*In utero* SHS exposure significantly increased mean linear intercept (L_m_) and decreased the surface area per unit volume (SApUV) of the lungs in both males and females. **a**) Data are expressed as mean ± standard error of the mean (SEM) (*n* = 5 per group). * *p* < 0.05 statistically different from the respective control group. **b**) 10× magnification of representative trichrome-stained slides of lung tissue of mice show that *in utero* SHS increases alveolar airspace of the lungs. *AM*: *in utero* air-exposed male mice; *SM*: *in utero* SHS-exposed male mice; *AF*: *in utero* air-exposed female mice; *SF*: *in utero* SHS- exposed female mice

We performed lung morphometric analysis on trichrome-stained lung tissues from SHS-exposed and air control mice to assess mean linear intercept (L_m_) and surface area per unit volume (SApUV), which can serve as indicators of lung tissue damage when respectively elevated and decreased [36]. Figure 2 demonstrates that *in utero* SHS exposure results in significantly increased L_m_ and significantly decreased SApUV, in both male and female mice at 15 weeks of age. *In utero* SHS exposure significantly increased L_m_ to 64 μm ± 5.9 versus 45 μm ± 2.2 for air-exposed males and to 60 μm ± 3.7 versus 45 μm ± 2.4 for air-exposed females (Fig. 2a). Figure 2b shows associated representative trichrome-stained slides of lung tissues. Airspace enlargement can be observed for SM and SF groups. Morphometric measurements of SApUV confirmed alterations in lung structure. The SApUV declined significantly for both male and female mice exposed *in utero* to SHS (37 ± 1.9 for SM and 39 ± 1.4 for SF, 1/mm) compared with their respective air-exposed controls (48 ± 2.2 for AM and 49 ± 2.3 for AF, 1/mm) (Fig. 2a).

### *In utero* SHS exposure alone decreases lung function in male mice

**Fig. 3:**
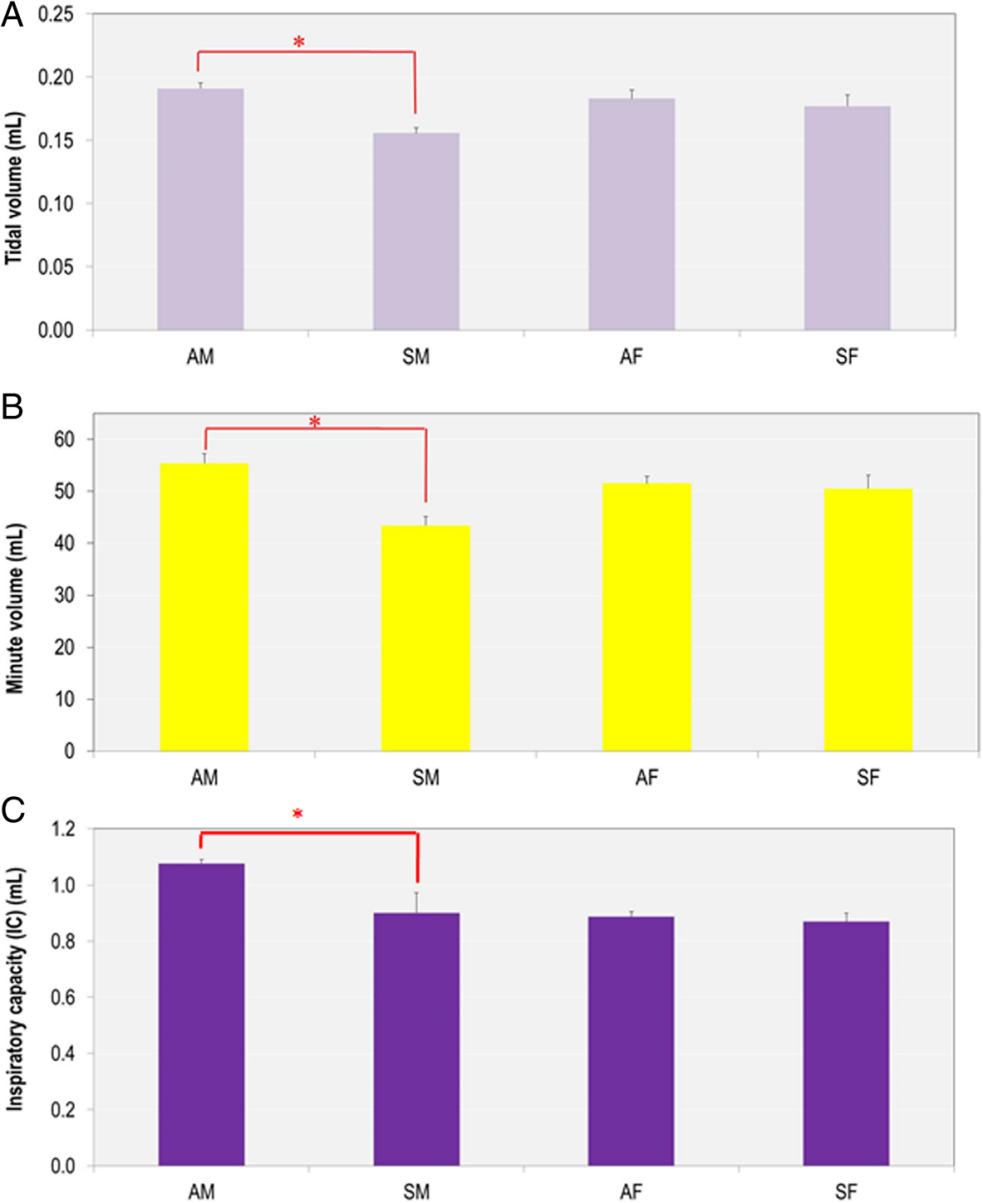
*In utero* SHS exposure significantly decreases tidal volume, minute volume and inspiratory capacity in 15-week old male mice. **a**) and **b**) data were measured by whole body plethysmography (*n* = 8 per group), and **c**) data were measured using a FlexiVent system (*n* = 6–7 per group). Data are expressed as mean ± SEM. *p < 0.05. *AM*: *in utero* air- exposed male mice; *SM*: *in utero* SHS- exposed male mice; *AF*: *in utero* air- exposed female mice; *SF*: *in utero* SHS-exposed female mice

Since *in utero* SHS exposure alone affected lung structure, we next asked whether these structural changes impacted lung function. At 15 weeks of age, pulmonary function testing at baseline levels revealed that tidal volume (0.191 ± 0.05 versus 0.156 ± 0.04 mL) and minute volume (55.44 ± 1.83 versus 43.42 ± 1.71 mL), measured by whole-body plethysmography, were significantly decreased (*p* < 0.05) in male mice exposed *in utero* to SHS compared with their respective air controls (Fig. 3a and b). Similarly, the inspiratory capacity, measured with the flexiVent system, showed a significant decrease (*p* < 0.05) for the lungs of male mice exposed *in utero* to SHS (1.08 ± 0.01 versus 0.90 ± 0.07 mL) (Fig. 3c). No significant differences were observed for these parameters in the female mice (Fig. 3). Since at 15 weeks of age there were no significant differences in the body weight of the mice exposed *in utero* to SHS or to filtered air (Table 2), the significantly reduced lung capacities (Fig. 3) that we observed in male mice exposed *in utero* to SHS were not due to body weight differences. In addition, we observed no indicators of pulmonary inflammation; the BALF of all groups were mainly composed of macrophages (data not shown), supporting the conclusion that decline in lung function in males was not related to obstructed airways caused by influx of leukocytes.

### *In utero* SHS exposure alone dysregulates the expression of genes and proteins in male mice

**Fig. 4:**
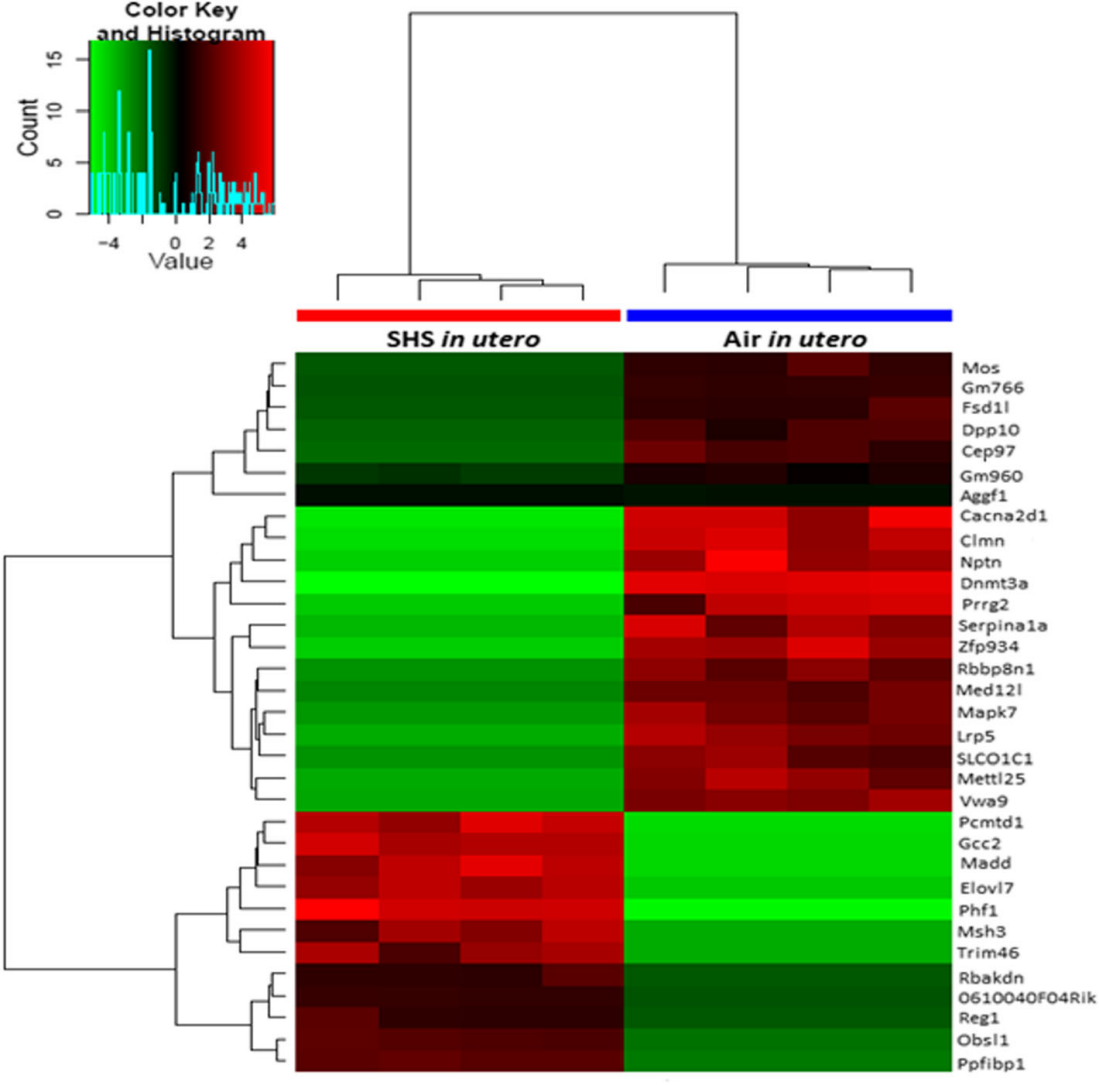
*In utero* SHS exposure dysregulates the expression of 33 genes, including functional clusters related to fibronectin, kinase and transcription regulation in 15-week old male mice. Results are presented for global gene expression of left lung (see methods for details regarding the gene expression analysis). Data are expressed as *in utero* SHS-exposed mice compared with air-exposed controls (log2 ratio fold increases) (*n* = 4 mice per group). Genes with at least 2-fold up-/down-regulation (*p* < 0.05) and FDR < 0.05 were considered differentially expressed

**Fig. 5:**
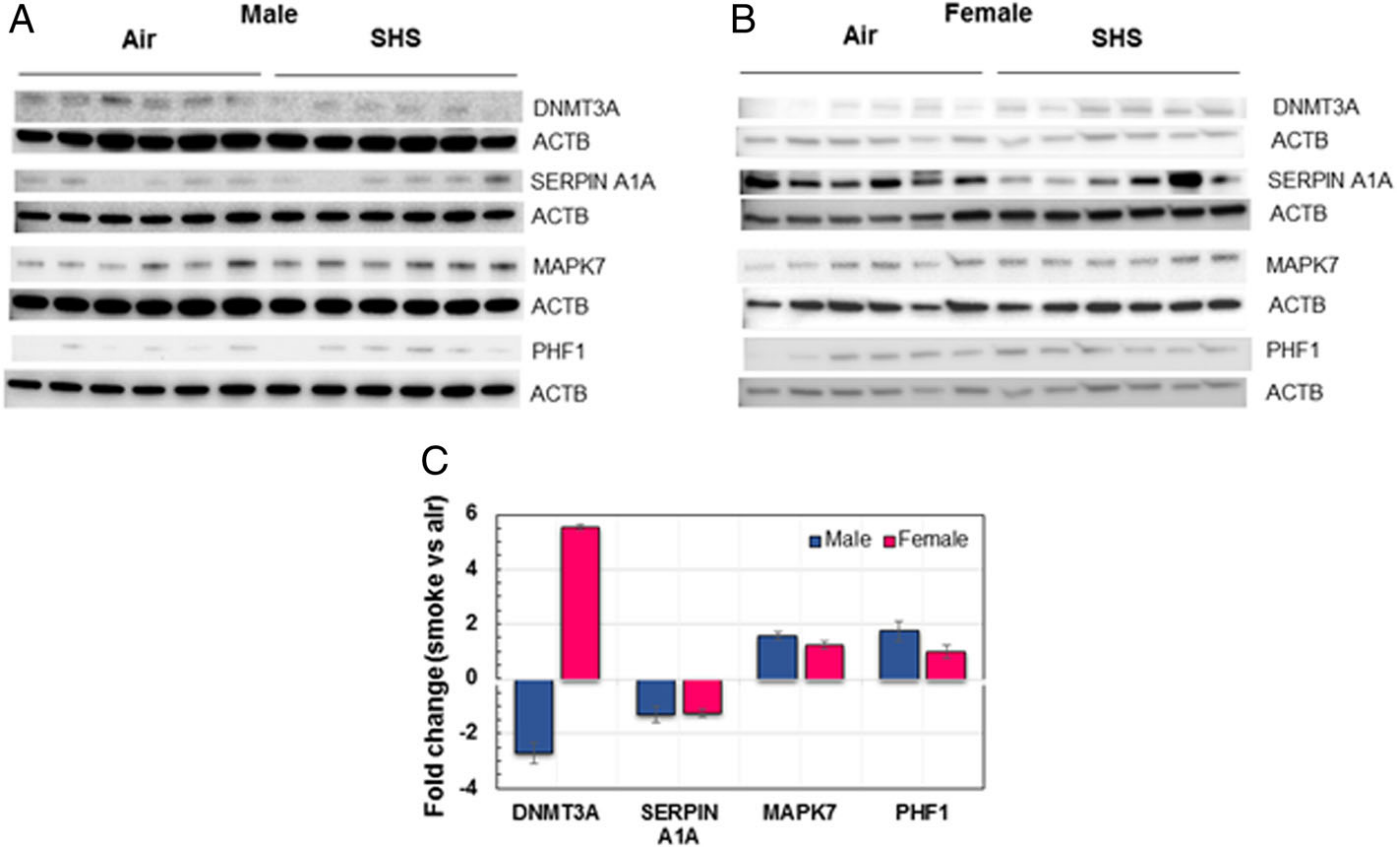
*In utero* SHS dysregulates the protein expression of DNMT3A, SERPINA1A, MAPK7, and PHF1 in male and female mice. **a**) Western blots show that DNMT3A and SERPINA1A were down-regulated, whereas MAPK7 and PHF1 were up-regulated in BALB/c male mice exposed *in utero* to SHS versus air-treated controls. **b**) Western blots show that SERPINA1A was down-regulated, whereas DNMT3A and MAPK7 were up-regulated in BALB/c female mice exposed *in utero* to SHS versus air-treated controls. **c**) Mean densitometry ± SEM results showing fold change of treated mice versus controls for the proteins analyzed

Next, to begin to address molecular mechanisms associated with the altered lung structure and decline in lung function we observed in male mice, we conducted RNA sequencing on lung tissue. This analysis was conducted only on male mice to identify the expression of key genes dysregulated by the *in utero* SHS exposure, since only male mice exhibited both structural and functional changes. Figure 4 shows that *in utero* SHS exposure in male mice dysregulated the expression of 33 lung genes, with 21 genes being down-regulated and 12 genes up-regulated. Nine of those genes were associated with three functional clusters: 1) a fibronectin type III (FN3) cluster [fibronectin type 3 and SPRY domain containing protein (*Fsd1l*), obscurin-like 1 (*Obsl1*), and tripartite motif-containing 46 (*Trim46*)]; 2) a kinase cluster [MAP-kinase activating death domain (*Madd*), Moloney sarcoma oncogene (*Mos*), and mitogen-activated protein kinase 7 (*Mapk7*)]; and 3) a transcription regulation cluster [DNA methyltransferase 3a (*Dnmt3a*), PHD finger protein 17 (*Phf1*), and mediator of RNA polymerase II transcription subunit 12 homolog (yeast)-like (*Med12l*)]. In addition, the gene *Serpina1a*, the mouse ortholog of the human gene α1-antitrypsin (*A1AT*), was down-regulated 7.3-fold in the SM mice compared with the AM controls. The Western blots (Fig. 5) confirmed the RNA sequencing results and were obtained for both male and female mice. Expression of both DNMT3A and SERPINA1A were down-regulated, while MAPK7 and PHF1 were up-regulated in male mice exposed *in utero* to SHS. Similar trends were found in female mice exposed *in utero* to SHS, with the exception of the DNMT3A protein which was up-regulated 5.5 fold compared with the air controls.

## Discussion

SHS is a major indoor air pollutant and increasing numbers of epidemiological and experimental studies have associated *in utero* exposure to SHS with adverse outcomes in newborns [2, 23, 25, 37–39]. Here we investigated whether *in utero* SHS exposure alone is sufficient to alter lung structure and function in adult mice. To the best of our knowledge, this is the first report of lung structural changes in adult mice exposed only to SHS *in utero*. We showed that *in utero* SHS exposure significantly increased Lm and decreased the SApUV of the lungs in both males and females (Fig. 2), indicating perturbation in alveolar developmental processes. Consequent to the lung structural alterations, the tidal volume, minute volume and inspiratory capacity were significantly decreased in male mice exposed *in utero* to SHS compared with the controls (Fig. 3), suggesting that males are more sensitive than females to an SHS insult during lung development. Additionally, the *in utero* SHS exposure dysregulated the expression of 33 genes (Fig. 4), including *Serpina1a*, the mouse ortholog of the human gene *A1AT*, with deficiency, or lower levels, of *A1AT* being known genetic risk factors for emphysema. Here *A1AT* was significantly down-regulated in SHS-exposed male mice. This suggests that *in utero* SHS exposure may predispose to emphysema development in adulthood. Furthermore, *Dnmt3a*, which is involved in DNA methylation, was significantly down-regulated at both gene (10 fold) and protein (2.7 fold) expression levels in SHS-exposed male mice (Figs. 4 and 5), suggesting a hypomethylated state of the lung tissue, which is known to be associated with activation of oncogenes [40–43]. In female mice, *in utero* exposures to SHS up-regulated the protein expression of DNMT3A 5.5 fold compared with air controls (Fig. 5), suggesting hypermethylation, which is related to silencing of tumor suppressors [40–43]. Overall, these data strongly indicate that *in utero* SHS exposure alone has significant persistent repercussions on the respiratory system, suggesting that *in utero* SHS exposure can predispose to at least some incurable adult lung diseases.

Morphometric measurements determined that, following *in utero* SHS exposure, both male and female mice at 15 weeks of age had altered lung structure, as evidenced by elevated L_m_ values, indicating 1) increased airspace size, and 2) diminished alveolar surface area (Fig. 2). Whether the observed SHS-induced alterations in lung structure in this mouse model are due to compromised alveolar development or to damaged septa walls is unknown [44]; however, the RNA-sequencing data suggest that *in utero* SHS affects extracellular matrix-associated gene expression, with the inclusion of 3 differentially expressed genes in the fibronectin type III cluster (Fig. 4). This cluster includes proteins involved in extracellular matrix remodeling (*Fsd1l*, *Obsl1* and *Trim46*; Fig. 4). A previous report noted that imbalances in the production of extracellular matrix proteins, including connective tissue growth factor, collagen and fibronectin, resulted in airway remodeling [45]. Moreover, *Serpina1a*, the mouse ortholog of the human gene *A1AT*, protects the lungs against elastase activity, which hydrolyzes proteins of the extracellular matrix, and thus contributes to lung structural breakdown [46]. *Serpina1a* was down-regulated at the gene expression level in the *in utero* SHS-exposed males and at the protein level in both male and female SHS-exposed mice (Figs. 4 and 5). At 15 weeks post-*in utero* SHS exposure, the expression of genes involved in lung development was not significantly dysregulated; however, at this time-point, expression of genes included in the fibronectin type III cluster was significantly changed (Fig. 4). This suggests that effects of *in utero* SHS on genes involved in the fibronectin type III cluster are important, regarding persistence of structural and functional alterations in adulthood. Overall, our data suggest that the airspace enlargement observed in the *in utero* SHS-exposed mice may be consequent to lung tissue remodeling, rather than solely subsequent to impaired lung growth and development. These results are supported by epidemiological studies and animal models, where it was previously shown that *in utero* cigarette smoke, as well as SHS, exposures affect the lungs of the offspring in terms of reduced growth, as well as enlarged alveoli that are present in fewer numbers (hypoalveolarization) [10, 25, 47–49]. Hence, in line with previous published reports, our study shows that *in utero* SHS exposure alone is a direct cause of alveolar structural defects, and therefore may increase the risk of developing respiratory diseases, including obstructive pulmonary diseases, e.g. emphysema, later in life.

Our study also showed that 15-week old male mice exposed *in utero* to SHS had significantly decreased lung capacities, including tidal and minute volumes, as well as inspiratory capacity (Fig. 3). These decrements in lung function seem to be secondary to changes in the lung architecture, as suggested by previous studies where *in utero* cigarette smoke-induced lung structural alterations impaired lung function by restricting airflow in small conducting airways [10, 25, 47–49]. In our model, it is unlikely that restricted airflow is responsible for the decreases in lung volumes, since *in utero* SHS had no significant effect on respiratory system resistance or Newtonian resistance (resistance of the conducting airways) at 15 weeks of age (data not shown). Since *in utero* SHS exposure alone significantly affected the lungs’ geometrical architecture, measured by alveolar airspace enlargement (Fig. 2), and gene and protein expression changes were largely associated with matrix airway-remodeling (Figs 4 and 5) in both male and female mice, with significant decline in lung function observed only in male mice (Fig. 3), the data suggest that changes in lung function may only be indirectly associated with lung structural damage. In fact, decline in lung function is multifactorial [50, 51] and our data suggest that lung structural changes may be necessary, but not sufficient, to impair lung function. Since all SHS fetuses were exposed simultaneously in the same chamber, these results also suggest that male mice are more susceptible than female mice to *in utero* SHS-induced damage, and that sex differences during lung development may play a pivotal role in healthy pulmonary function.

It is well recognized that for many mammalian species, including mice and humans, critical factors in lung development, including surfactant production and fetal breathing, are events beginning earlier in the lung maturation process of female fetuses compared with males [52–56]. Lung surfactant synthesis provides the pulmonary phenotype with adequate air flows, resistance and compliance, and thus plays a key role in optimal pulmonary function and lung homeostasis [56–60]. This suggests that the synthesis of surfactant occurring at an earlier developmental time point in females may protect the lungs against environmental insults, such as SHS, with regard to lung function. Epidemiological studies support this sex bias, with females being preferentially protected from early lung environmental irritants. The prevalence for wheeze and asthma is higher in boys than in girls [61–65]. These findings correlate with results of a previous study from our laboratory showing that male mice exposed *in utero* to SHS exhibited enhanced asthmatic responses following ovalbumin treatment compared with their female counterparts at 23 weeks of age [23]. Whether there is potential for lung recovery following *in utero* SHS exposure cannot be determined from our study since we only analyzed one time-point. In accordance, however, with the results of the present study, epidemiological evidence showed declines in lung function in offspring exposed *in utero* to cigarette smoke or SHS in childhood [8, 66–73]. These declines were sustained until early adulthood (21 years) in males [74]. This suggests that even though there may be some lung recovery, *in utero* cigarette smoke or SHS pulmonary effects persist into childhood and early adulthood. This study provides additional data demonstrating that lung function alterations that are sustained through early adulthood seem to be sex-specific, with males being more susceptible than females to *in utero* SHS effects; thus, potentially increasing the risk of males to develop respiratory diseases, including restrictive lung diseases.

Overall, we showed that even at 15 weeks of age, the effects of *in utero* SHS exposure can be seen in terms of altered lung structure and function, as well as altered gene and protein expression in male mice (Figs 2, 3, 4 and 5). The exact mechanisms linking the *in utero* SHS exposure to those adverse lung effects in adulthood are unknown; however, it is widely recognized that several types of environmental exposures, including air pollution [75], maternal diet [76], and cigarette smoke [77], induce epigenetic alterations that could impact lung development, as well as lung repair following acute injury [78]. Epigenetic mechanisms can modify the epigenome in a transitory manner or permanently into adulthood [79]. DNA methylation regulates gene expression through an epigenetic silencing mechanism and is involved in tumorigenesis by influencing, among others, the expression of oncogenes or tumor suppressor genes [41]. DNA (Cytosine-5-)-Methyltransferase 3 Alpha (Dnmt3a), an enzyme that catalyzes the transfer of methyl groups to specific CpG structures in DNA, is responsible for *de novo* DNA methylation, and thus that plays a central role in this mechanism [74]. The methylation status of DNA can either be hyper- or hypomethylated, although global DNA hypomethylation and specific locus hypermethylation frequently co-occur in human cancers [40, 42]. Numerous studies [80–83] showed that DNA methylation has roles in both early and late stages of tumorigenesis. In early stages, increased levels of DNA methylation lead to hypermethylation of tumor suppressor genes and thus, facilitate tumor initiation. In late stages, decreased levels of DNA methylation lead to hypomethylation of oncogenes, which promotes cancer progression [43]. Our RNA-sequencing results, confirmed by protein expression (Figs. 4 and 5), indicate that *in utero* SHS exposure alone significantly down-regulated (10.4 fold change at the gene level and 2.7 fold change at the protein level; Figs. 4 and 5) the expression of Dnmt3a in males, which suggests the possibility of DNA hypomethylation of other lung genes. This hypomethylation finding is supported by an epidemiological study conducted by Breton et al. [77] that used buccal and aerodigestive tissue cells (as surrogates for lung cells) of children exposed *in utero* to cigarette smoke and showed that this exposure resulted in significantly decreased global methylation, i.e. hypomethylation, as well as gene-specific DNA methylation alterations [77]. Global DNA hypomethylation is associated with development of cancer [84], including lung cancer, which has been associated with Dnmt3a deficiency in mice [85, 86]. Furthermore, in our study, while *in utero* SHS-exposed male mice exhibited down-regulation of the DNMT3A protein (2.7 fold), female mice that underwent the same treatment showed up-regulation of this protein by 5.5 fold (Fig. 5). In a previous study, it was observed that methylation levels can be influenced by sex, although no mechanisms have been proposed [40]. As mentioned previously, it is well known that both types of alteration are associated with tumor formation, with hypomethylation status being associated with activation of oncogenes and hypermethylation with silencing of tumor suppressors [40]. Thus, in addition to our results, there is increasing evidence for epigenetic effects of *in utero* SHS exposure alone on lung development with persistence into adult life, and this may predispose to respiratory morbidity, including lung cancer [39].

## Limitations

One limitation of this study is that RNA-sequencing was not performed on female mice, whose *in utero* SHS-exposed group exhibited only significant structural differences compared with controls. Although RNA-sequencing is a powerful tool to understand the transcriptome of cells under specific conditions, it can be very difficult to detect differences between control and treatment groups when the treatment (or insult) occurred 15 weeks earlier, even though the physiological responses are significant. We have previously showed that upon re-exposure to SHS as adults, mice exposed *in utero* to SHS exhibited altered lung structure and dysregulation of some genes, as detected by microarray assays [21]. Since only male mice exhibited both structural and functional changes in this study, we conducted RNA-sequencing to add valuable molecular mechanism insights for those significant differences. Therefore, based on the RNA sequencing results obtained in male mice, we analyzed the protein expression change of key affected molecules to further explore the molecular mechanisms in both sexes. Changes at the protein level are sensitive and were mostly consistent (Fig. 5) between males and females. Thus, in this study, for the female mice, we used protein expression as a proxy to obtain a better understanding of the molecular signatures imprinted by *in utero* SHS.

Another possible limitation of this study is the fact that mice were sacrificed at only one time-point. As mentioned previously, whether there is potential for lung recovery or whether lung tissue remodeling is more important than impaired growth and development following *in utero* SHS exposure cannot be determined from our study since we only analyzed the lungs 15 weeks post-exposure. It is important, however, to bear in mind that the objective of this study was to investigate whether *in utero* SHS exposure alone, without any postnatal re-exposure to an irritant, is sufficient to alter lung structure and function in adult 15-week old mice. Studies are currently ongoing in our laboratories to investigate *in utero* and neonatal developmental effects induced by SHS, hookah smoke and electronic cigarette vapor.

## Conclusions

This study shows that *in utero* SHS exposure alone induces a wide range of effects on the developing lung. These changes persist in adult male mice at least through 15 weeks of age. Alterations included changes in lung structure and function, accompanied by dysregulation of genes and proteins involved in extracellular matrix-related airway remodeling and DNA methylation. Our findings also indicate that male mice may be more susceptible than female mice to insults occurring during lung development. Overall, this study provides baseline data indicating that *in utero* SHS exposure alone imprints the developing lung for potential lifelong sequelae that predispose the lung to adult obstructive and restrictive respiratory diseases, as well as lung cancer. More research is needed to provide insight on the contribution of *in utero* SHS exposures to the pathophysiology of emphysema and lung cancer development.

## Abbreviations

ACTB: β-actin
AF: Air *in utero* females
AM: Air *in utero* males
BAL: Broncho-alveolar lavage
BALF: Broncho-alveolar lavage fluid
EPA: Environmental Protection Agency
FDR: False discovery rate
Lm: Mean linear intercept
PAH: Polynuclear aromatic hydrocarbon
qRT-PCR: Quantitative real-time polymerase chain reaction
SApUV: Surface area per unit volume
SD: Standard deviation
SEM: Standard error of the mean
SF: SHS *in utero* females
SHS: Second-hand smoke
SM: SHS *in utero* males
WHO: World Health Organization
